# Leprosy in children under 15 years of age in a municipality in northeastern Brazil: evolutionary aspects from 2003 to 2015

**DOI:** 10.1590/0037-8682-0515-2020

**Published:** 2020-11-25

**Authors:** Pedro Martins Lima, Antônio Rafael da Silva, Leonardo Hunaldo dos Santos, Raina Jansen Cutrim Propp Lima, Pedro Luiz Tauil, Eloísa da Graça do Rosário Gonçalves

**Affiliations:** 1 Universidade Federal do Maranhão, Programa de Pós-Graduação em Saúde e Ambiente, São Luís, MA, Brasil.; 2 Universidade Federal do Maranhão, Centro de Ciências Sociais, Saúde e Tecnologia, Imperatriz, MA, Brasil.; 3 Universidade Federal do Maranhão, Centro de Referência em Doenças Infecciosas e Parasitárias, Departamento de Patologia, São Luís, MA, Brasil.; 4 Instituto Federal de Educação, Ciência e Tecnologia do Maranhão, Departamento de Ensino, Açailândia, MA, Brasil.; 5 Universidade de Brasília, Programa de Pós-Graduação em Medicina Tropical, Brasília, DF, Brasil.

**Keywords:** Leprosy, Mycobacterium leprae, Epidemiology

## Abstract

**INTRODUCTION:**

The Integrated Program of Leprosy Control was initiated in 2003 in the municipality of Buriticupu, Maranhão, Brazil, an area considered hyperendemic for leprosy. Here, we present the evolution of the indicators of leprosy within the established period in children aged <15 years.

**METHODS:**

This is a descriptive study based on an active search for cases and spontaneous healthcare demand for leprosy, with an evolutionary analysis of the detection coefficient of new cases. We considered individuals aged <15 years diagnosed with leprosy from January 2003 to December 2015. To evaluate the factors associated with clinical and operational forms, Chi-square, Fisher’s exact, or Fisher-Freeman-Halton tests were performed.

**RESULTS:**

A total of 61 new cases were detected (6.9% of the total leprosy cases diagnosed in the municipality during the study period), and the majority was found in males (62.3%). The most frequent operational classification was paucibacillary (67.2%), and this association increased with age. The tuberculoid clinical form was the most prevalent in both sexes and in the age range of 10 to <15 years. There was a reduction in the detection coefficient from 21.84/100,000 inhabitants in 2003 to 2.79/100,000 in 2015.

**CONCLUSIONS:**

Despite the progress in the control of leprosy, this historical series shows that it is necessary to strengthen educational measures and implement control actions, so that the disease ceases to be a public health problem in the population aged <15 years.

## INTRODUCTION

Leprosy is a chronic infectious disease whose etiological agent is *Mycobacterium leprae* and is most often transmitted by close contact with infected and untreated people^1^. The main clinical manifestations are skin and neurological lesions, which can cause physical disabilities and psychosocial limitations^2^.

Leprosy is the oldest known human disease. According to Robbins et al.^3^, archaeological records from 2000 B.C., during the Chalcolithic age, evidenced the appearance of the disease. Based on the historical context, it is assumed that no other disease caused as much stigma and social exclusion as leprosy due to its association with punishments, impurities, and sins. Lack of knowledge about the disease and fear of disease carriers are feelings that may delay the search for treatment^4^.

In 1990, the World Health Organization (WHO) proposed a global goal to eliminate leprosy by the end of the 20th century. Despite the commitment of governments, researchers, and health workers, worldwide control of the disease was not achieved. However, by the end of the year 2000, 107 countries had successfully eliminated leprosy^5^. In 2016, a new strategy for eliminating the disease by 2020 was proposed, with the main objectives being: reducing the number of children diagnosed with leprosy and presenting visible physical deformities to zero, all countries enacting specific legislation against discrimination, and reduction of new leprosy cases with grade-2 disability to less than one case per million^6^.

In Brazil, leprosy is still an important public health problem as it is the second country with the highest number of new cases notified, accounting for 92.3% of cases from the American continent in 2017^7^. In the same year, Brazil had a prevalence of 1.35 cases per 10,000 inhabitants, corresponding to 28,067 cases under treatment. The general coefficient of detection of new cases of leprosy was 12.94 per 100,000 inhabitants, corresponding to 26,875 new cases of the disease, which characterizes a classification of high endemicity according to official parameters. Of these, 1,718 were among children aged <15 years, representing a detection coefficient of 3.72 per 100,000 inhabitants. Early exposure and persistent transmission of the disease in younger age groups are important for assessing its prevalence, contributing to the perception of an endemic pattern of leprosy in a given location^8^.

Although it is rare in children, particularly in those aged less than five years^5^, the speed at which leprosy spreads and its clinical presentation depend on host characteristics, etiological agent, and unfavorable social determinants such as quality of life, sanitation, and high concentration of people in the same dwelling place^1^. The number of leprosy cases in those aged <15 years is an important indicator for determining the level of transmission of the disease, and it indicates the need to intensify or implement specific prevention and control measures for this age group^9^.

This study was oriented toward a demographic, epidemiological, and clinical interpretation of leprosy in a given period in the population of the municipality of Buriticupu, State of Maranhão, Brazil. Leprosy in this municipality has been characterized as an important public health problem, evidencing historical problems that go along with the disease, such as social stigma, vulnerabilities due to the patients’ socioeconomic situation, precarious organization of local health services, and lack of analysis of the epidemiological indicators of the disease, which could serve as a support to guide the actions of the municipality administrators and health care professionals. This study aimed to assess the epidemiological characteristics of the disease in children aged <15 years, which is an important factor in eliminating the disease.

## METHODS

This is a descriptive study of a case series based on the diagnosis of leprosy by active search for cases and spontaneous healthcare demand, an evolutionary analysis of the detection coefficient, and the number of new cases from January 2003 to December 2015. The data were obtained from the database of the Center for Studies in Tropical Medicine of the Federal University of Maranhão, in the municipality of Buriticupu, located in the western Maranhão state, in the pre-Amazon Maranhão region.

The following parameters were considered for the diagnosis of leprosy: changes in skin color or sensitivity and bacilloscopy of skin smears performed for all patients and read according to the formula established by Ridley and Jopling in 1962^10^. Whenever doubts about the definition of the clinical form, histopathological examination of the lesions was performed.

Sociodemographic and clinical information was studied based on the following variables: age group (1-4, 5-9, and 10 to <15 years), sex, operational classification (paucibacillary or multibacillary) as proposed by the WHO in 1982^11^, clinical form, and place of origin of the case. Clinical forms were defined as indeterminate, tuberculoid, dimorph, and lepromatous according to the Madrid classification^12^. For the calculation of the detection coefficient, the population <15 years of age, from the same place and period in which new cases of the disease were diagnosed, was considered each year. 

The collected data were stored in a specific database created in Microsoft Excel, version 2016 (Software Foundation, Inc., Boston, MA, USA). After purging the errors and inconsistencies, a descriptive analysis was performed using relative and absolute frequencies of sociodemographic and clinical characteristics. To evaluate the factors associated with the clinical and operational forms, Chi-square tests with and without Yates continuity correction, Fisher’s exact, or Fisher-Freeman-Halton tests^13^ were performed. For significant associations, we also estimated odds ratios, considering a 95% confidence interval. All tests were performed using SPSS version 24^14^(IBM, Armonk, NY, USA) at 5% significance.

In compliance with the requirements of Resolution 466/2012 of the Brazilian National Health Council, this study was approved by the Research Ethics Committee (Approval No. 234,767, protocol No. 12700713.9.0000.5084).

## RESULTS

The analysis and processing of the data elucidated to a great level of detail the epidemiological and clinical profiles of leprosy in the municipality of Buriticupu during the study period, when 879 new cases of the disease were diagnosed. In children aged <15 years, 61 new cases were diagnosed, representing 6.9% of the total. Among these, 62.3% were male and 86.9% of the diagnosed patients were aged between 10 and 15 years. Regarding the clinical profile, 67.2% of patients were classified as paucibacillary and 44.2% had the tuberculoid clinical form. Autochthonous cases represented most diagnoses (93.4%) (Table 1).

**TABLE 1:** Sociodemographic and clinical characteristics of children with leprosy aged <15 years in Buriticupu, Maranhão, in 2003-2015.

Variables	N	%
**Gender**		
Male	38	62.3
Female	23	37.7
**Age group (years)**		
5-9	8	13.1
10 to <15	53	86.9
**Operational classification**		
Paucibacillary	41	67.2
Multibacillary	20	32.8
**Clinical form**		
Indeterminate	14	23.0
Tuberculoid	27	44.2
Dimorph	12	19.7
Lepromatous	8	13.1
**Case source**		
Autochthonous	57	93.4
Imported	4	6.6
**Total**	**61**	**100**

Source: Author’s data (2020).

Table 2, which demonstrates the distribution of cases by sex in relation to the operational classification, shows a predominance of males in both the multibacillary and paucibacillary forms. In all age strata, there was a predominance of the paucibacillary form, and this proportion increased with age. Regardless of the operational classification, the age group with the largest number of cases was 10 to <15 years, with 53 cases. Statistical analysis showed no association between sex (p = 0.16) or age group (p = 0.92) and operational classification.

**TABLE 2:** Cases of leprosy in children aged <15 years by sex, age group, and operational classification in Buriticupu, Maranhão, in 2003-2015.

Operational classification						
	Paucibacillary		Multibacillary		Total	*p-value*
	N	%	N	%		
Gender						0.16
Male	23	60.5	15	39.5	38	
Female	18	78.3	5	21.7	23	
**Age group (years)**						
5-9	6	75.0	2	25.0	8	0.92
10 to <15	35	66.0	18	34.0	53	

Chi-square test. Source: Author’s data (2020).

Although the clinical form showed no statistical association in relation to sex (p = 0.56), there was a higher proportion of tuberculoid cases, particularly in females (52.2%; Table 3).

**TABLE 3:** Cases of leprosy in children aged <15 years by clinical form and sex in Buriticupu, Maranhão, in 2003-2015.

	Gender					
Clinical form	Male		Female		Full	*p-value*
	N	%	N	%		
Indeterminate	8	21.1	6	26.1	14	0.56
Tuberculoid	15	39.4	12	52.2	27	
Dimorph	9	23.7	3	13.0	12	
Lepromatous	6	15.8	2	8.7	8	

Chi-square test. Source: Author’s data (2020).

There was no association between age groups and clinical forms (p = 0.76; Table 4). However, the data show that in all clinical forms, there were a higher number of cases in the age group between 10 to <15 years, especially for the tuberculoid form, which represented 45.2% of the cases in this age group.

**TABLE 4:** Cases of leprosy in children aged <15 years by clinical form and age group in Buriticupu, Maranhão, in 2003-2015.

Age group (years)						
Clinical forms	5-9		10 to <15		Full	*p-value*
	N	%	N	%		
Indeterminate	3	37.5	11	20.8	14	0.76
Tuberculoid	3	37.5	24	45.2	27	
Dimorph	1	12.5	11	20.8	12	
Lepromatous	1	12.5	7	13.2	8	

Chi-square test. Source: Author’s data (2020).

**FIGURE 1:**
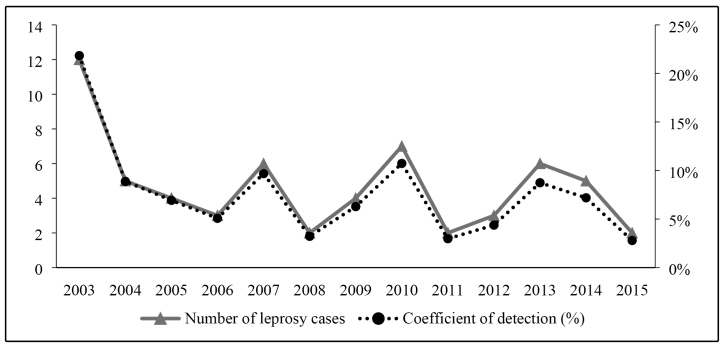
Historical series of detection coefficients and number of cases in children with leprosy aged <15 years in the Municipality of Buriticupu, State of Maranhão, Brazil, from 2003 to 2015.

The number of cases and the annual detection coefficient for new cases per 100,000 inhabitants in children aged <15 years showed a decreasing trend from 2003 to 2015 **(** Figure 1 **)**. When analyzing the detection coefficient using the endemicity parameters, it can be concluded that 2003 and 2010 were hyperendemic (21.84 and 10.73/100,000 inhabitants, respectively), 7 years (2004, 2005, 2006, 2007, 2009, 2013, and 2014) had very high endemicity, and 4 (2008, 2011, 2012, and 2015) had high endemicity.

## DISCUSSION

Although leprosy is considered to be a disease of adults and young adults, there are many cases in age groups <15 years, and this is an important indicator for determining the level of transmission of the disease^15^. Data published by the Brazilian Ministry of Health in 2015 indicate a detection rate of 7.3% in children aged <15 years^16^. This value was similar to that found in the current study (6.9%). Leprosy cases in children aged <15 years indicate early exposure to the bacillus, recent transmission of the disease, operational precariousness of primary-care surveillance, and little active case search action. These factors favor maintenance of the endemic status of the disease and become sensitive elements for evaluating its dimension^17^.

The finding of paucibacillary forms in young individuals aged <15 years shows that the municipality of Buriticupu is an area of expansion of the disease, with active transmission by maintaining bacilliferous sources and a persistence of late diagnosis^18^ ^,^ ^19^.

Regarding the clinical classification, it is desirable to find the largest number of cases in the indeterminate clinical form because they present a better prognosis^1^. However, there was an average proportion of this form of the disease (23%), and according to Silva et al.^18^, these results may be related to delayed diagnosis, probably because primary-care centers fail to detect cases at the onset of the disease.

In the present study, the tuberculoid form was the most frequent (44.2%), similar to the results of a survey conducted from 2003 to 2012 by Souza, Rocha, and Lima^19^ in 132 patients in the Brazilian city of Juazeiro, State of Bahia, which corresponded to 62.1% of cases. According to Luna, Moura, and Vieira^20^, the presence of the tuberculoid form is an important epidemiological indicator of an increasing trend of leprosy, characterized as a form of resistance.

The results of the present study showed that in children aged <15 years 32.8% cases were multibacillary, despite the tendency to decrease and stabilize. This type of notification in this age group may reflect an early exposure to risk contacts and a high density of sources of infection^21^. The increase in the detection coefficient within a historical series may be a sign of worsening of the endemic condition, a warning sign of change in the pattern of transmission from community contact to household contact, or even a classification error.

In the present study, the detection coefficient in children aged <15 years showed a strong decrease during the study period, in contrast to what was observed by Souza and Rodrigues^22^, who showed that in the period from 2002 to 2012, there was no significant decline (5.56/100,000, in 2012) in their analysis of a time series in this age group.

Other studies have indicated a constant trend over time^20^. In Brazil, there was a significant decline in the burden of the disease. In the data from northeastern Brazil, as of 2015, the disease is still considered to have high endemicity in the entire country (3.72/100,000 inhabitants) and very high endemicity in the Northeast (6.19/100,000 inhabitants). The global annual detection of leprosy has shown a tendency to decline in all age groups since 2001^6^.

Further, regarding the evolutionary aspects of the endemic disease, there was a decrease of approximately 87.2% in the reduction rates in 2015 relative to 2003. This indicates a decrease in the sources of infection, possibility of transmission control, and increased possibilities of cure without sequelae. A survey conducted from 2002 to 2011 in the State of Maranhão also reported a significant decrease in the infection rates of leprosy^23^. Other studies, despite reporting fewer cases in this age group, did not report such high rates^19^ ^,^ ^20^. Therefore, a decrease in these indices is an important step toward reducing and controlling the disease in the municipality.

The present study has some limitations, especially in data collection, with missing information, which compromised the input of other sociodemographic and clinical variables. As this is a cross-sectional study, we highlighted the possibility of providing rapid feedback to the population. In addition to contributing to its field of knowledge, it is hoped that this study can stimulate discussions among the region administrators, social representatives, and health teams, so that new goals for the control and future elimination of leprosy in the municipality are achieved.

Despite improvements in the coverage of the family health strategy and an increased number of healthcare facilities and professionals, the municipality of Buriticupu needs to strengthen and consolidate its health services. For this, investments must be made in expanding the active search for cases, appropriate examination of household contacts, and the execution of educational campaigns.
